# Prevalence of human papillomavirus in head and neck cancers in European populations: a meta-analysis

**DOI:** 10.1186/1471-2407-14-968

**Published:** 2014-12-17

**Authors:** Seye Abogunrin, Gian Luca Di Tanna, Sam Keeping, Stuart Carroll, Ike Iheanacho

**Affiliations:** Evidera Inc., Metro Building, 6th Floor, 1 Butterwick, London, W6 8DL UK; Statistical Advisor to Evidera Inc., Metro Building, 6th Floor, 1 Butterwick, London, W6 8DL UK; Sanofi Pasteur MSD, Mallards Reach, Bridge Avenue, Maidenhead, SL6 1QP UK

**Keywords:** HPV, Human papillomavirus, Head and neck cancer, Prevalence

## Abstract

**Background:**

Infection with human papillomavirus (HPV) is necessary for the development of cervical carcinoma. By contrast, the role of HPV in the pathogenesis of other malignancies, such as head and neck cancers, is less well characterised. This study aimed to address key information gaps by conducting a systematic review and meta-analysis of the prevalence of HPV infection in head and neck cancers, focusing on data for European populations.

**Methods:**

MEDLINE, Embase and grey literature sources were systematically searched for primary studies that were published in English between July 2002 and July 2012, and which reported on the prevalence of HPV infection in head and neck cancers in European populations. Studies on non-European populations, those not published in English, and those assessing patients co-infected with human immunodeficiency virus were excluded. Eligible studies were combined in a meta-analysis. In addition, the potential statistical association between the head and neck cancers and certain HPV types was investigated.

**Results:**

Thirty-nine publications met the inclusion criteria. The prevalence of HPV of any type in 3,649 patients with head and neck cancers was 40.0% (95% confidence interval, 34.6% to 45.5%), and was highest in tonsillar cancer (66.4%) and lowest in pharyngeal (15.3%) and tongue (25.7%) cancers. There were no statistically significant associations between the HPV types analysed and the geographical setting, type of sample analysed or type of primer used to analyse samples in head and neck cancers.

**Conclusions:**

The prevalence of HPV infection in European patients with head and neck cancers is high but varies between the different anatomical sites of these malignancies. There appears to be no association between HPV type and geographical setting, type of samples analysed or type of primer used to analyse samples in such cancers.

**Electronic supplementary material:**

The online version of this article (doi:10.1186/1471-2407-14-968) contains supplementary material, which is available to authorized users.

## Background

Recent evidence suggests that, in Europe, the incidence and mortality of cancer of the oral cavity are 99.6 per 100,000 population and 44.3 per 100,000 population, respectively [1]. Moreover, the World Health Organization has estimated that, across the continent, the 5-year prevalence of cancers of the lip, oral cavity and pharynx is over 250,000 cases [2]. These data are part of a global disease picture in which, each year, around 600,000 people develop some form of head and neck cancer and around 300,000 die from it [3]. This condition’s diverse clinical spectrum and the associated burden of illness, has fuelled interest in potential aetiological factors and the extent to which they can be prevented or modified. While risks such as tobacco use and alcohol consumption are widely recognised carcinogens for head and neck cancers, the role of the human papillomavirus (HPV) in this setting has received much less attention, until recently.

HPV can infect the stratified epithelia of the skin or mucous membranes of the upper gastrointestinal, respiratory or ano-genital tract, potentially leading to outcomes such as genital warts and laryngeal papillomas, as well as certain cancers. The association with cancers has led to the various types of HPV being termed ‘low-risk’ or ‘high-risk’ depending on their known oncogenic potential [4]. In general, the worldwide incidence and prevalence rates of HPV-related cancers have been rising, with studies suggesting that the risk of developing these conditions increases with the number of lifetime sexual partners [5–7]. HPV’s oncogenic role is most clearly defined in cervical cancer, in which the virus is a necessary pathogenic factor. By comparison, its aetiological contribution to the other malignancies is less well characterised. For instance, HPV is associated with only a subset of head and neck cancers [8], with various reviews estimating that the virus is detectable in approximately 12.8% − 59.9% of all head and neck squamous cell carcinoma biopsies [9–12].

HPV-16 and HPV-18 are the predominant types found in HPV-related cancers and are the main focus of current vaccination programmes in European countries, aimed at reducing the occurrence of HPV infection and related cervical cancer. These programmes have been based on vaccines that are bivalent, targeting high-risk types with regards to oncogenic potential (HPV-16 and HPV-18), or quadrivalent targeting both low-risk (HPV-6 and HPV-11) and high-risk types. However, such programmes could be extended to target other oncogenic types (HPV-31, HPV-33, HPV-45, HPV-52, and HPV-58), which are included in new higher-valency vaccines. It is worth noting that available literature for European populations has not yet quantified to what extent the burden of illness for various head and neck cancers is caused by high-risk HPV types. In addition, previous analyses of the benefits or cost-effectiveness of vaccination programmes in Europe have not, in general, accounted for cancers in sites other than the cervix [13–18].

Indeed, few recent publications have systematically reviewed and pooled data on the overall prevalence of HPV infections in head and neck cancers, and assessed the presence of the specified oncogenic HPV types (i.e. HPV etc.). Also, of five published meta-analyses that attempted to quantify the prevalence of HPV in head and neck cancers [9–12, 19], only two reported overall pooled HPV prevalence estimates for a European population [9, 11].

The need for additional evidence on the relationship between HPV and head and neck cancers is highlighted by the increasing interest stakeholders in Europe have shown in this topic. For example, the European Commission is funding major epidemiological and clinical research in this area [20] and the role of HPV has become a key theme of scientific conferences on head and neck cancers [21, 22]. Also, HPV-related oral cancer has been debated recently in the United Kingdom (UK) Parliament [23] and the UK Joint Committee on Vaccination and Immunisation (JCVI) has established a HPV sub-committee, whose remit will include examining the case for extending the national HPV vaccination programme to help prevent head and neck cancers associated with the organism [24, 25].

Against this background, the current study aimed to systematically review published studies to quantify the prevalence of HPV types (specifically, 6, 11, 16, 18, 31, 33, 45, 52, and 58) in head and neck cancers, as documented within published studies on European populations and, thereby, address key evidence gaps.

## Methods

### Literature search

**Figure 1:**
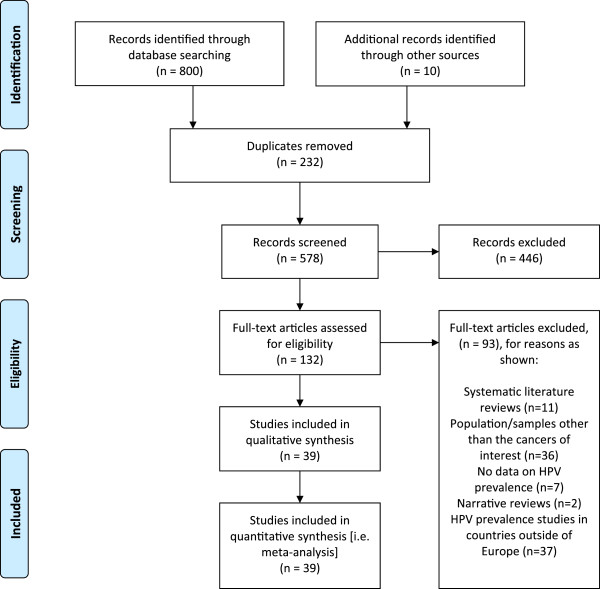
**PRISMA Diagram showing flow of literature through search and screening process.**

MEDLINE, Embase and grey literature sites were systematically searched for potentially relevant primary studies published between 2002 and 2012, by using words synonymous with ‘human papillomavirus’, ‘HPV’, ‘prevalence’, and ‘cancer’, combined with terms representing regions of the head and neck (Additional file 1: Table S1 and S2). The search terms for the anatomical regions were deliberately broad because of the known variation in how the sites of origin of HPV-related head and neck cancers have been termed across different publications (a lack of standardisation that impedes research in this field). Publications identified through the searches were screened using a two-step process, comprising initial title and abstract screening to select publications for subsequent full-text screening. At both steps, each publication for screening was reviewed by two researchers using pre-defined inclusion criteria (Additional file 2: Table S1). Disagreements between the researchers on the inclusion of particular publications were resolved through discussion with a third researcher. Figure 1 shows the flow of literature through the search and screening process.

### Data extraction and quality assessment

For each study included in the systematic review, a researcher extracted data on the prevalence of HPV types of interest (6, 11, 16, 18, 31, 33, 45, 52, and 58) in all the head and neck cancers studied, with a view to the potential inclusion of this information in subsequent meta-analyses. The accuracy of the data extraction was then checked by a second researcher and, again, any discrepancies between the researchers were resolved through discussion with a third researcher.

Each study included in the systematic review was rated for scientific quality by two independent researchers using a modified version of the Methodological Evaluation of Observational Research (MORE) criteria. This grading tool was deemed most suitable as it was developed specifically for epidemiological studies of chronic diseases [26] and would account for the methodological robustness of the studies in assessing the prevalence of HPV. However, the original MORE criteria was selectively adapted because almost none of the publications reported information relevant to the criteria ‘inter-rater reliability’ and ‘validation of the assessment/measurement methods’, and so would have automatically been graded as low quality, thus, reducing the sensitivity of the tool. Therefore, these two criteria were omitted and only those remaining (the ‘modified MORE criteria’) were applied in rating the studies for quality. Further details of the MORE criteria are provided in Additional file 2: Table S2).

### Meta-analytic approach

A feasibility assessment was conducted to determine whether there was sufficient evidence from the included studies to undertake a classical meta-analysis on the prevalence of HPV in the cancers of interest. Having confirmed that such an analysis was justifiable, this approach was then used to generate a pooled estimate of prevalence of HPV, with a 95% confidence interval (CI), for the head and neck cancer sites of interest, both collectively and as individual sites. Prevalence estimates were calculated as the number of total patients (or samples) infected with the HPV types of interest divided by the number of patients (or samples) evaluated.

Oral and mouth cancers were treated as a single category and this meant we analysed 10 separate head and neck cancer categories (Table 1). Some of the data reported in six studies were deemed unclassifiable, mainly because it was not possible to separate the head and neck cancers studied into constituent cancer types. [27–32]. Four of these publications reported some classifiable data [27, 29, 30, 32]. The other two studies, with no such data, were included only in the overall pooled prevalence for head and neck cancers [28, 31]. Subgroup analysis was performed only on the analysable data in these publications. As all data used in the analysis were taken from published sources, no ethics committee approval was sought.

**Table 1:** **Number of included studies by type of cancer**

Cancer	Number of included studies^a^
Base of tongue	5
Hypopharyngeal	5
Laryngeal	10
Oral	18
Oropharyngeal	14
Paranasal sinus	1
Pharyngeal	4
Tongue	4
Tonsillar	13
Waldeyer’s ring	2
Unclassifiable	6

^a^Some of the articles reported more than one type of cancer and presented the data for each type separately, allowing the data for each type to be regarded as a separate study. As such, the total number of articles based on classification by cancer type does not equal the total number of included studies in the systematic review and meta-analysis.

### Statistical methods

If the extracted HPV prevalence estimates are regarded as constituting a random sample from a larger population of studies, then meta-analysis of those data can be viewed as a survey [33] in which one first obtains a random sample of studies from a larger population of such studies, and then from within each of these selected studies one obtains a random sample of subjects from the population. This two-stage sampling can be represented mathematically as follows [34]:


Where

Var is variance,

T_i_ is an estimate of effect size (the prevalence) for study *i,*

θ_i_ is the true effect size for study *i*,

e_i_ is the deviation between Ti and θ_i_ (caused by ‘sampling error’),

τ is the random-effects variance,

σ_i_^2^ is the estimation, or fixed-effects variance for study *i.*

These equations mean that if there is no random variation in the prevalence (effect size) from study to study, τ = 0, and all variation between the estimated prevalence from different studies can be attributed to variation within the sample included in the study (so-called ‘sampling error’). This is the assumption made by so-called fixed-effects meta-analytical (FEM) approaches. By contrast, random-effects meta-analytic (REM) approaches allow for the possibility that random variation between studies accounts for some of the variation between their results. We conducted the meta-analysis of HPV prevalence using both the FEM and REM approaches because the estimates generated were expected to reflect the presence of heterogeneity between the studies [35].

Each REM analysis was used to estimate the mean of (and standard error for) θ_i_; estimate τ; test the hypothesis τ = 0 using Cochran’s Q statistic I^2^, a measure of the proportion of the overall variability between study estimates caused by true heterogeneity between studies [36].

### Investigation of heterogeneity

Heterogeneity between the studies in this systematic review might be explained by several factors [37], including differences in study design; patient populations; patient/sample inclusion and exclusion criteria across studies; HPV DNA source (the type of histological sample used to test for the presence of HPV DNA), polymerase chain reaction (PCR) primers used to confirm presence of the virus; and other methodological features. Cancerous tissues can be tested for the presence of HPV infection by either examining exfoliated cells (saline washings; saline brushing or tissue scrapings) or fixed-biopsy samples (formalin-fixed paraffin embedded biopsy samples; fresh biopsy samples or frozen fixed biopsy samples), using various DNA detection techniques, such as DNA/RNA microarray, histoimmunohistochemical staining, in-situ hybridisation, p16 immunostaining, polymer chain reaction with or without in-situ hybridisation, signal amplification and southern blot assay methods [38]. Therefore, to try to explain the quantitative heterogeneity found in the pooling of the prevalence estimates, exploratory meta-analyses were conducted across the following variables (chosen because of the availability of sufficient data on these characteristics across the included studies):

- Type of samples analysed
  - ○ Fixed biopsy
  - ○ Others (‘other types of samples’)
- Types of primers used for the analysis
  - ○ GP5+/GP6+ combinations
  - ○ MY09/11 combinations (not including GP5/GP6); others (‘other primers’)
- Geographical location of patient population or samples
  - ○ Eastern Europe
  - ○ Western Europe

### Exploration of statistical association between the head and neck cancers and each HPV type

Additionally, meta-regressions were performed to determine whether any of the HPV types were particularly associated with certain categories of head and neck cancer. As cancer categories with fewer studies would not have had enough data to be analysed separately, the following groups were investigated: laryngeal, oral, oropharyngeal and tonsillar cancers, with the other head and neck cancers categorised as ‘others’, for the purposes of this exploration.

Statistical analysis was performed with STATA software (StataCorp. 2009. Stata Statistical Software: Release 12. College Station, TX: StataCorp LP). The raw data used in the meta-analysis are available from the authors on request.

## Results

### Descriptive overview of included studies

Our systematic review identified 568 abstracts from the search of indexed databases (MEDLINE and Embase), once 232 duplicates had been removed. From these 568 unique citations, the full texts of 122 articles were identified and studied for their relevance to the review, using the inclusion criteria described in Additional file 2: Table S1, in addition to 10 articles identified from grey literature sources. Of these, 39 articles reported on the prevalence of the pre-specified HPV types in head and neck cancers. Further details of included studies can be found in Additional file 2: Table S3).

All 39 of the publications reporting on the prevalence of HPV types in one or more types of head and neck cancer [27–32, 39–71] related to cross-sectional or other observational studies. Seventeen of the articles evaluating patients or samples of patients with head and neck cancer reported on multiple cancer types and presented the data on each type separately, allowing each of these datasets to be regarded as a separate study [27, 30, 32, 40, 41, 43, 44, 50, 51, 53–56, 58, 60, 67, 71]. Table 1 shows the frequency of included studies by cancer type.

Owing to the substantial heterogeneity between the studies (I^2^: 96.3%; τ: 0.0536), sources of patient/sample populations were categorised as being in either a Western or an Eastern European location (see Table 2), and meta-analysed on that basis. In general, each article included in our analysis reported on patients or samples from only one European country. Three articles, however, reported on patients from multiple European countries [39, 52, 57], while the geographical location of the study population or samples was unclear in one article [54]. Table 3 shows the distribution of included articles by country.

**Table 2:** **Geographical classification of source of patient population/samples**

Western Europe	Eastern Europe
Austria, Denmark, England, Finland, France, Germany, Greece, Hungary, Italy, Norway, The Netherlands, Portugal, Scotland, Spain, Sweden and the United Kingdom (UK)	Czech Republic, Lithuania, Poland, Slovenia and Turkey

**Table 3:** **Number of included articles by country source(s) of patients/samples**

Country source(s) of patients/samples	Number of articles
Czech Republic	3
Denmark	1
Finland	1
Finland, Norway and Sweden	1
France	1
Germany	9
Germany and Greece	1
Hungary	4
Italy	7
Lithuania	1
The Netherlands	1
Norway, Sweden and the UK	1
Poland	1
Scotland	1
Slovenia	1
Sweden	2
Turkey	1
UK	1
Unclear	1
**Total**	**39**

Fixed biopsy was the method most commonly used to collect cancer samples to assess the prevalence of HPV DNA, being reported as the sole collection technique in 32 articles [27–32, 42–45, 47–53, 55, 57–70]. Two articles reported collection of cancer samples using exfoliating methods [39, 46], while one study used both fixed-biopsy and exfoliating methods [41], and one used serum samples alone [71]. Three studies did not report the sample collection method [40, 54, 56].

### Quality assessment (using modified MORE criteria)

Most studies included in this systematic review were graded as Level 1B and 2B (moderate to poor quality), according to the modified MORE Levels of Evidence ratings (scale 1A–2C, with 1A representing the highest level of evidence) (see Table 4).

**Table 4:** **Overview of quality ratings for primary studies identified in the systematic review and meta-analysis**

Modified MORE rating^a^	Modified MORE levels of evidence definition	Number of studies^b^
1A (Good)	Fewer than 4 major flaws, plus 0–1 minor flaws	3
1B (Moderate)	Fewer than 4 major flaws, plus 2–3 minor flaws	17
1C (Moderate)	Fewer than 4 major flaws, plus 4 or more minor flaws	5
2A (Poor)	4 or more major flaws, plus 0–1 minor flaws	4
2B (Poor)	4 or more major flaws, plus 2–3 minor flaws	6
2C (Poor)	4 or more major flaws, plus 4 or more minor flaws	4

^a^TA Shamliyan, RL Kane, MT Ansari, G Raman, ND Berkman, M Grant, G Janes, M Maglione, D Moher and M Nasser [72].

^b^The count of publications in this table includes primary studies.

### Prevalence of HPV in head and neck cancers

REM estimates should be regarded as the primary results of the analysis because of the substantial heterogeneity amongst the included studies. With this approach, the overall pooled prevalence of HPV (including types 6, 11, 16, 18, 31, 33, 45, 52 and 58), as determined by the presence of viral DNA, in head and neck cancers was 40.0% (95% CI, 34.6% to 45.5%). Estimates by type of cancer indicated that the prevalence of HPV infections was highest in tonsillar cancer (66.4%; 95% CI, 57.2% to 75.6%). The systematic review also identified two eligible studies reporting on cancers of Waldeyer’s ring (a broader anatomical category including various tonsillar and tonsil-like tissues), the pooled prevalence of HPV for this group was estimated to be 32.9% (95% CI, 12.7% to 53.1%) [32]. By contrast, HPV prevalence estimates were lowest for pharyngeal cancer (15.3%; 95% CI, 3.0% to 27.7%), and next lowest for tongue cancer (25.7%; 95% CI, 3.4% to 47.9%). Only one study reporting on HPV types in paranasal sinus cancer met the inclusion criteria and this found the prevalence of HPV infection in this type of cancer to be 60.3% (95% CI, 20.6% to 100.0%)^a^.

**Figure 2:**
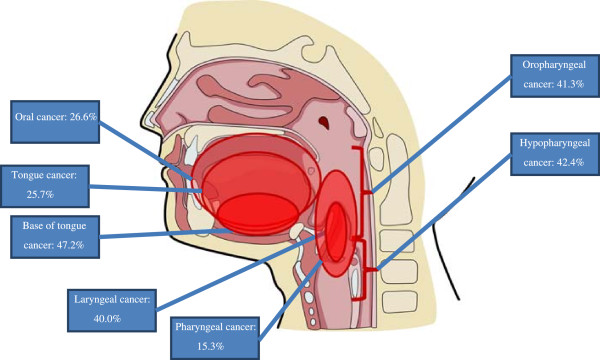
**Prevalence of HPV in cancers in various head and neck regions.**

Table 5 summarises the overall prevalence of HPV and number of patients/patient samples analysed by cancer type. Figure 2 is a graphical presentation of the prevalence of HPV by cancer site.

**Table 5:** **Prevalence of HPV by head and neck cancer type** ^**a**^

Cancer type	Number of patients/patient samples tested	Prevalence (%)	95% confidence interval	
			Low (%)	High (%)
Base of tongue	193	47.2	37.3	57.1
Hypopharyngeal	50	42.4	14.7	70.2
Laryngeal	498	40.0	34.6	45.5
Oral	1,157	26.6	19.8	33.3
Oropharyngeal	894	41.3	31.8	50.7
Pharyngeal	25	15.3	3.0	27.7
Tongue	113	25.7	3.4	47.9
Tonsillar	605	66.4	57.2	75.6
Waldeyer’s ring	113	32.9	12.7	53.1
All head and neck cancers	3,649	40.0	34.6	45.5

^a^Aggregate head and neck cancer prevalence estimates include one case of paranasal sinus cancer with unadjusted prevalence estimates of 100.0%; 95% CI: 60.3% to 139.7% (adjusted values to reflect prevalence estimates exceeding 100%: 60.3%; 95% CI: 20.6% to 100.0%).

### Relationships between HPV prevalence and potential sources of study heterogeneity

In general, there was a lack of statistically significant associations between the prevalence of HPV infection in head and neck and potential sources of study heterogeneity, such as type of cancer sample analysed, type of DNA primer used to detect the virus or geographical location.

### Geographical location of patient population or samples by cancer type

The only statistically significant finding with regards to geographic location was identified in oropharyngeal cancers, for which HPV prevalence was statistically lower for Western European populations (33.7%; 95% CI, 24.2% to 43.2% vs. Eastern countries 56.9%; 95% CI, 49.5% to 64.3%). The prevalence of HPV infection in four of the cancer groups (base of tongue, oral, pharyngeal and tonsillar) was numerically, but not significantly, lower for populations in Western Europe than in Eastern Europe. The prevalence was numerically higher in tongue cancers among Western European than among Eastern European populations but, again, not significantly. Additional data from the investigation by geographical location are provided in Table 6.

**Table 6:** **Summary of HPV prevalence by source of heterogeneity (Prevalence; 95% confidence interval)**

Type of cancer	Geographic location			Type of samples analysed			Type of primer used			
	Eastern Europe	Western Europe	Unclear	Fixed-biopsy	Others	Not reported	GP5+/GP6+	MY09/MY11 (not GP5+/GP6+)	Others	Not reported
Base of tongue	50.0%; 95% CI, 23.7% to 76.3%	44.5%; 95% CI, 29.4% to 59.5%	53.7%; 95% CI, 40.9% to 66.6%	41.5%; 95% CI, 26.9% to 56.1%	Not applicable	53.7%; 95% CI, 44.6% to 62.8%	50.0%; 95% CI, 23.7% to 76.3%	Not applicable	39.5%; 95% CI, 20.2% to 58.8%	53.7%; 95% CI, 44.6% to 62.8%
Hypopharyngeal	Not applicable^a^			Not applicable^b^			60.0%; 95 CI%, 20.3% to 100.0%^c^	50.0%; 95 CI%, 21.5% to 78.5%	48.9%; 95 CI%, 0.0% to 97.9%^d^	29.3%; 95 CI%, 12.0% to 46.3%
Laryngeal	27.5%; 95 CI%, 0.0% to 55.1%^c^	28.0%; 95 CI%, 14.8% to 41.2%	Not applicable	32.0%; 95 CI%, 17.0% to 47.1%	19.7%; 95 CI%, 12.8% to 26.6%	Not applicable	25.6%; 95 CI%, 5.1% to 46.0%	56.0%; 95 CI%, 0.0% to 100.0%^c,d^	29.6%; 95 CI%, 4.7% to 54.7%	20.5%; 95 CI%, 14.0% to 26.9%
Oral	34.7%; 95 CI%, 9.4% to 60.0%	21.3%; 95 CI%, 15.0% to 27.7%	Not applicable	27.5%; 95% CI, 20.3% to 34.8%	26.6%; 95% CI, 19.8% to 33.3%	Not applicable	34.8%; 95 CI%, 17.6% to 52.0%	34.5%; 95 CI%, 14.2% to 54.8%	16.9%; 95 CI%, 0.0% to 34.6%^c^	16.7%; 95 CI%, 0.0% to 34.6%^c^
Oropharyngeal	56.9%; 95% CI, 49.5% to 64.3%	33.7%; 95% CI, 24.2% to 43.2%	Not applicable	39.6%; 95% CI, 29.7% to 49.5%	33.3%; 95% CI, 20.4% to 46.2%	64.0%; 95% CI, 55.4% to 72.7%	46.8%; 95 CI%, 33.2% to 60.3%	36.5%; 95 CI%, 0.0% to 73.1%^c^	37.8%; 95 CI%, 15.5% to 60.2%	37.7%; 95 CI%, 19.9% to 55.5%
Pharyngeal	20.0%; 95% CI, 0.0% to 49.4%^c^	14.3%; 95% CI, 0.7% to 27.9%	Not applicable	Not applicable^b^			29.4%; 95 CI%, 0.0% to 58.8%^c^	29.4%; 95 CI%, 0.0% to 58.8%^c^	22.1%; 95 CI%, 0.0% to 44.1%^c^	Not applicable
Tongue	12.2%; 95% CI, 0.0% to 24.4%	32.1%; 95% CI, 0.9% to 63.2%	Not applicable	34.2%; 95% CI, 0.0% to 68.3%	21.4%; 95% CI, 10.9% to 32.0%	Not applicable	12.2%; 95 CI%, 0.0% to 24.4%^c^	Not applicable	33.3%; 95 CI%, 0.0% to 100.0%^c,d^	21.4%; 95 CI%, 10.9% to 32.0%
Tonsillar	80.4%; 95% CI, 69.7% to 91.1%	64.2%; 95% CI, 54.1% to 74.3%	Not applicable	65.0%; 95% CI, 54.2% to 75.8%	Not applicable	73.3%; 95% CI, 65.6% to 81.1%	62.4%; 95 CI%, 43.5% to 81.3%	80.3%; 95 CI%, 68.2% to 92.4%	62.6%; 95 CI%, 30.0% to 95.1%	68.7%; 95 CI%, 61.6% to 75.8%

^a^All studies investigated patients from Western Europe.

^b^The sources of all cancer types were fixed biopsies.

^c^Adjusted values to reflect prevalence estimates less than 0.0%.

^d^Adjusted values to reflect prevalence estimates exceeding 100%.

### Type of samples analysed by cancer type

The estimated prevalence of HPV in oropharyngeal cancers was significantly lower in studies that used fixed-biopsy samples (39.6%; 95% CI, 29.7% to 49.5%) and other types of samples (33.3%; 95% CI, 20.4% to 46.2%) than in the only study not reporting the type of samples analysed (64.0%; 95% CI, 55.4% to 72.7%). The prevalence of HPV was numerically, but not significantly, lower in studies using fixed-biopsy samples of base of tongue and tonsillar cancers than in those that did not report the type of samples analysed. However, the prevalence in oral, laryngeal and tongue cancers was numerically, but not significantly, higher in fixed-biopsy samples than in other types of samples. Additional data from the investigation by sample type are provided in Table 6.

### Types of primers used for HPV DNA analysis by cancer type

There were no significant findings in relation to the types of primers used for HPV DNA analysis by cancer type. The prevalence of HPV was numerically, but not significantly, lower in studies of tongue and tonsillar cancer that had analysed HPV DNA types using only a primer that included GP5+/GP6+ than in studies using alternative primers. Also, the prevalence of HPV was numerically, but not significantly, lower in studies of oral cancers that analysed HPV DNA types either using other types of primers or those that did not report the primers used than those using GP5+/GP6+ primers. Additionally, HPV prevalence was numerically, but not significantly, lower in pharyngeal cancer studies that assessed HPV DNA types using other type primers than in those that used either GP5+/GP6+ or MY09/MY11 primers. Additional data from the investigation by primer type are provided in Table 6.

### Meta-regression of pooled prevalence estimates of HPV types by covariates

Meta-regressions were possible only for pooled prevalence estimates of the following HPV types: 6, 11, 16, 18 and 33. The covariates in these analyses were European regional location used as covariates. As shown in Table 7, none of the meta-regressions showed any statistically significant association between the HPV types and the covariates.

**Table 7:** **Pooled prevalence estimates of HPV types by covariate** ^**a**^

HPV type	Covariates	Coefficient	Standard error	95% confidence interval		p-value
				Low	High	
HPV-6	Western vs. Eastern	−0.059	0.198	−0.609	0.492	0.782
	GP5+/GP6 + ^b^	0.124	0.171	−0.350	0.599	0.507
	Laryngeal^c^	0.040	0.136	−0.393	0.473	0.788
	Oral^c^	0.459	0.210	−0.208	1.126	0.116
HPV-11	Western vs. Eastern	−0.005	0.090	−0.393	0.383	0.960
	Fixed biopsy vs. others	−0.018	0.142	−1.825	1.788	0.919
	GP5+/GP6 + ^b^	0.017	0.118	−1.478	1.512	0.908
	MY09/MY11^b^	0.032	0.169	−2.112	2.176	0.882
	Laryngeal^c^	−0.014	0.160	−2.009	1.981	0.944
	Oral^c^	0.006	0.129	−1.634	1.647	0.968
HPV-16	Western vs. Eastern	0.101	0.100	−0.105	0.308	0.320
	Fixed biopsy vs. others	0.356	0.282	−0.228	0.939	0.220
	GP5+/GP6 + ^b^	−0.095	0.108	−0.321	0.132	0.393
	MY09/MY11^b^	−0.066	0.161	−0.403	0.271	0.687
	Laryngeal^c^	−0.171	0.155	−0.493	0.152	0.283
	Oral^c^	−0.030	0.131	−0.303	0.242	0.820
	Oropharyngeal^c^	0.183	0.133	−0.094	0.460	0.184
	Tonsillar^c^	0.194	0.126	−0.069	0.457	0.139
HPV-18	Western vs. Eastern	0.249	0.302	−0.713	1.210	0.471
	Fixed biopsy vs. others	0.191	0.407	−1.103	1.485	0.671
	GP5+/GP6 + ^b^	0.207	0.477	−1.847	2.261	0.707
	MY09/MY11^b^	−0.039	0.592	−2.587	2.509	0.954
	Laryngeal^	−0.146	0.398	−1.414	1.121	0.737
HPV-33	Western vs. Eastern	−0.029	0.032	−0.099	0.040	0.375
	GP5+/GP6 + ^b^	−0.039	0.042	−0.132	0.056	0.390
	MY09/MY11^b^	0.036	0.157	−0.314	0.386	0.825
	Laryngeal^c^	0.017	0.094	−0.197	0.230	0.863
	Oral^c^	−0.082	0.084	−0.272	0.108	0.354
	Oropharyngeal^c^	−0.094	0.070	−0.251	0.064	0.212
	Tonsillar^c^	−0.108	0.068	−0.262	0.045	0.144

^a^Significance level: p < 0.05.

^b^Compared with ‘other’ types of primers (not including GP5+/GP6+ or MY09/MY11 combinations).

^c^Compared with ‘other’ cancers (base of tongue, hypopharyngeal, paranasal sinus, pharyngeal, tongue, unclassifiable, and Waldeyer’s ring).

## Discussion

The aim of this systematic review and meta-analysis was to provide up-to-date information on the associations between the presence of HPV and the various head and neck cancers in European populations, and factors that might influence these relationships. Specifically, we wanted to generate estimates of the prevalence of HPV infection in the key sites of head and neck cancer. The results of the analysis indicated that the prevalence of HPV in such disease was high overall (at around 40%) but also varied considerably between the different malignancies, the pooled prevalence estimates for oral cancers (18 studies), laryngeal cancer (10 studies), oropharyngeal cancer (14 studies) and tonsillar cancer (13 studies), being 26.6%, 25.7%, 41.3% and 66.4%, respectively. All these findings should help to address important gaps in the literature.

The systematic review and meta-analysis were based on data retrieved from 39 articles reporting on the prevalence of HPV infections in head and neck cancers in European populations and published between 2002 and 2012. This means it offers more specific and up-to-date information than previous reviews on this topic. Of the other five known published meta-analyses that have quantified the prevalence of HPV in head and neck cancers, the first (including studies published up to 2004) reported only on the prevalence of HPV-16 [19]; the second included studies published up to 2004, reporting on a total of 37 HPV types [9]; the third (and the only other to report on a European population) estimated overall pooled HPV prevalence but only from studies published up to 2010 [11]; the fourth included only studies that reported on HPV-16 and HPV-18 (published between 1980 to 2008) [10] and the fifth included studies up to 2007 and examined 12 HPV types [12]. While the dataset appears to be more up-to-date than that of other reviews, further primary studies assessing HPV prevalence in non-cervical cancers in European populations continue to be published, and should ideally be incorporated in further reviews and meta-analyses on this topic.

As expected, all studies included in this review and meta-analysis were observational in design and most analysed populations or samples from Western (n = 16) rather than Eastern (n = 5) Europe, according to our geographical classification. The available data related primarily to the more common forms of head and neck cancer, such as disease involving the oral cavity, oropharynx and larynx. By contrast, there was only one study of cancer of the paranasal sinus (based on a single case from a publication on a broader group of patients with head and neck cancer), suggesting that this rare form of head and neck cancer is comparatively under-researched with respect to any association with HPV. Overall, the studies included in the analysis were of poor to moderate quality according to the modified MORE criteria.

In contrast to previous studies [9–12, 19], our analysis extended the search period for evidence collection to 2012, and identified more recent studies of Eastern European populations [28, 48, 55, 63], compared with another meta-analysis which did not identify data in articles published after 2005 [11]. Even so, the high overall prevalence of HPV infection in head and neck cancer derived through our analysis is similar to that reported in a recently published meta-analysis on European populations and an older global estimate (34.5% and 39.7%, respectively) [11, 12]. Also, while the prevalence of HPV infection across the different categories varied widely, similar estimates to those in our study have been reported in other meta-analyses for populations in Europe [9, 11]. In addition, the prevalence of HPV in oropharyngeal cancers in our study was similar to that in other meta-analyses, and lower than that found among North American populations in those studies (47.0% [9] and 59.9% [11], respectively).

To some extent, the wide range of HPV prevalence between different cancers in our study might reflect variations in the amount of data available and, therefore, the precision of estimates for particular cancers. In addition, not only was the number of available studies very limited for some of the head and neck cancer categories, there was considerable heterogeneity among included studies, potentially contributing to the overall wide range of HPV prevalence. However, even allowing for such sources of variation in the prevalence data, there still appeared to be marked differences between those cancers for which more data were available and the estimates therefore more precise (as suggested by their narrower CIs). Such evidence does not establish categorically that there are differences between head and neck cancers with regards to HPV prevalence, for example, those suggested between oral or pharyngeal cancers, and oropharyngeal cancers. It is possible that the results for these latter regions were random findings from heterogeneous data, and that actual prevalence for the oropharyngeal region is somewhere in the range of 20%–40%. Further research is required to clarify this issue. However, pending this, it is important to note again that previous studies have also described differences in HPV prevalence between various site-specific carcinomas [9, 12, 73].

Equally, however, these results caution against automatically assuming that HPV has a similar pathogenic role in these anatomically linked conditions, even though the differences in prevalence between sites were not statistically significant. There are, of course, other risk factors associated with particular head and neck regions that may increase the overall risk of oncogenesis in these regions. For example, smoking is more likely to affect paranasal sinuses [74], and alcohol plus tobacco consumption the tongue and oral cancers [75, 76]. Previous work on HPV prevalence and head and neck cancers has stated the need for further research to clarify the virus’ role in such conditions, including any co-interactions with other carcinogenic factors, such as smoking [73, 77]. Such research is needed to determine whether HPV prevalence overestimates HPV's true contribution to development of these cancers.

A secondary objective of this study was to perform meta-regression to seek potential associations between the presence of particular HPV types and pre-defined covariates in the different categories of head and neck cancer. Overall, these analyses provided no clear evidence for or against such associations. This included inconclusive results from meta-regressions investigating possible associations between HPV prevalence in these cancers and the type of sample used to identify the presence of the organism. These findings are interesting given that existing evidence has suggested that the use of exfoliated cells to identify HPV infection might be an unreliable method that has low detection rates and, therefore, gives a misleadingly low indication of prevalence when compared with fixed-biopsy samples [8, 78].

The meta-analysis of the prevalence of HPV infection in different head and neck cancers had various limitations. Our analysis did not include all oncogenic and non-oncogenic HPV types. However, our results provide up-to-date data on HPV types 6, 11, 16, 18, 31, 33, 45, 52 and 58. Another limitation of our study was the lack of high-quality studies for inclusion in the meta-analysis, which resulted in the substantial heterogeneity identified within the available data set. In view of the latter, we tested potentially influential study covariates that may have accounted for differential prevalence estimates in the HPV types identified in the different cancers, in addition to analysing the data using fixed- and random-effects models. However, this additional exploration did not provide definitive evidence as to source(s) of the heterogeneity. Other potential sources of heterogeneity not investigated in our meta-analysis included the date the cancer samples were taken, which could provide data on changes in HPV prevalence in the countries evaluated. This could be also important if, over time, demographic and other risk factors associated with head and neck cancers had changed to different degrees in different countries. Another consideration not accounted for in this analysis was the time it takes to develop cancer, since variations in the duration between risk factor acquisition and cancer diagnosis could have added to heterogeneity.

The meta-regression analyses used in the study had the potential to explore important associations between HPV and different head and neck cancers, but were also associated with considerable limitations. These included difficulties relating to the meta-regression methods used to pool prevalence estimates of HPV types by country, types of samples analysed and primer types. To conduct this analysis, the three independent factors of interest (country, types of samples analysed and primer types) were considered to be categorical. Clear categories for the various head and neck cancers were initially sought after. However, these efforts were complicated by the lack of information on how individual authors defined the regions of the head and neck in their studies. Ultimately, therefore, the classifications reported in each of the selected articles had to be used within our analysis.

The meta-regression approach was also limited as it is particularly suited for outcomes that are continuous and have values within a potentially non-limited range; this was clearly not the case for the covariates examined in our study. Another relevant issue with the meta-regression approach is that the standard errors of the prevalence estimates (i.e., the inverse of the weights) are equal to 0 when the HPV prevalence for a particular study is 0 or 1; and this leads to the automatic exclusion of that specific study from the analysis. A potential alternative analytical approach in this context would be the use of logistic regression to investigate the presence of any associations between prevalence estimates and the different cancer types. However, as with the meta-regression used in the current study, the robustness of any results could still be threatened by the lack of data available for some of the required analyses. Finally, it is important to note that estimation of the prevalence of various HPV types in each of the categories of the head and neck cancer was beyond the intended scope of our study. Further research on this topic in European populations would be a key addition to the literature.

Current deliberations among researchers and policymakers in Europe, such as those of the UK Parliament and the JCVI HPV sub-committee, show how important and timely it is to increase understanding of the disease burden associated with HPV. This need is particularly pressing for head and neck cancers in the UK, where significant epidemiological changes in these malignancies are being observed [79]. In this context, we believe that the results of our analysis provide key data on the prevalence of HPV infection in head and neck cancer in European populations, information likely to be of interest to a wide range of stakeholders.

## Conclusions

This study indicates that the prevalence of HPV infection in people with head and neck cancers in Europe is high but appears to vary widely between the different cancer types. Considerable heterogeneity was found across the studies obtained. However, there was no clear evidence of an association between geographical setting, type of samples analysed or type of primer, and HPV type in such malignancies.

## Endnote

^a^Adjusted values reflect prevalence estimates exceeding 100%, original values (100.0%; 95% CI: 60.3% to 139.7%).

## Electronic supplementary material

Additional file 1: **Search Strategy.** (DOCX 21 KB)

Additional file 2: Table S1: Inclusion and Exclusion Criteria. **Table S2**. Modified Methodological Evaluation of Observation Research (MORE) Grading Criteria. **Table S3**. Characteristics of Included Studies. (DOCX 43 KB)

## Abbreviations

AIDS: Acquired immunodeficiency syndrome
CI: Confidence interval
FEM: Fixed-effects meta-analytical
HIV: Human immunodeficiency virus
HPV: Human papillomavirus
JCVI: Joint Committee on Vaccination and Immunisation
MORE: Methodological Evaluation of Observational Research
REM: Random-effects meta-analytical
UK: United Kingdom.
