# Supplementary material for: Prevalence of human papillomavirus in head and neck cancers in European populations: a meta-analysis
Source: BMC Cancer. 2014 Dec 17;14:968. doi: 10.1186/1471-2407-14-968 (PMC4320477; doi:10.1186/1471-2407-14-968)
Supplement: Supplementary file 1 — Additional file 1: Search Strategy. (DOCX 21 KB) [file 12885_2014_5178_MOESM1_ESM.docx]

# Additional file 1:

# Search Strategy

**Indexed database search**

We reviewed MEDLINE-indexed publications using the following search strategy. The limits for this search included: only items with abstracts, humans, English and published between July 2002 and July 2012.

Table S1. MEDLINE

|  | String | Search Terms | Yield |
| --- | --- | --- | --- |
| #1 | HPV String | "human papillomavirus"[Title/Abstract] OR "human papilloma virus"[Title/Abstract] OR hpv*[Title/Abstract] OR papilloma[Title/Abstract] OR papillomavirus[Title/Abstract] | 35098 |
| #2 | Disease String | penis[Title/Abstract] OR penile[Title/Abstract] OR anus[Title/Abstract] OR anal[Title/Abstract] OR vagina*[Title/Abstract] OR vulva* [Title/Abstract] OR oral[Title/Abstract] OR oropharyn*[Title/Abstract] OR head[Title/Abstract] OR neck[Title/Abstract] OR mouth[Title/Abstract] OR tongue[Title/Abstract] | 817461 |
| #3 | Prevalence String | epidemiology[Title/Abstract] OR prevalence[Title/Abstract] | 398234 |
| #4 | Cancer String | cancer*[Title/Abstract] OR carcinoma*[Title/Abstract] OR malignanc*[Title/Abstract] OR neoplasm*[Title/Abstract] | 1343278 |
| #5 |  | #1 AND #2 AND #3 AND #4 | 773 |
| #6 | Not Reviews String | (review[pt] NOT (systematic OR meta-analy* OR ((indirect OR mixed) AND "treatment comparison"))) | 1562995 |
| #7 |  | #5 NOT #7 | 629 |
| #8 |  | #7; Limits: English, Publication Date from 2002 to 2012 | 357 |

We have also limited the search to not include non-systematic reviews published prior to 2002.

We reviewed EMBASE-indexed publications using the following search strategy. The limits for this search included: only items with abstracts, humans, English and published July 2002 and July 2012.

Table S2. EMBASE

|  | String | Search Terms | Yield |
| --- | --- | --- | --- |
| #1 | HPV String | 'human papillomavirus':ti OR 'human papilloma virus':ti OR hpv*:ti OR papilloma:ti OR papillomavirus:ti OR 'human papillomavirus':ab OR 'human papilloma virus':ab OR hpv*:ab OR papilloma:ab OR papillomavirus:ab AND [humans]/lim AND [english]/lim AND [abstracts]/lim AND [embase]/lim AND [2002-2012]/py | 13,564 |
| #2 | Disease String | penis:ti OR penile:ti OR anus:ti OR anal:ti OR vagina*:ti OR vulva*:ti OR oral:ti OR oropharyn*:ti OR head:ti OR neck:ti OR mouth:ti OR tongue:ti OR penis:ab OR penile:ab OR anus:ab OR anal:ab OR vagina*:ab OR vulva*:ab OR oral:ab OR oropharyn*:ab OR head:ab OR neck:ab OR mouth:ab OR tongue:ab AND [humans]/lim AND [english]/lim AND [abstracts]/lim AND [embase]/lim AND [2002-2013]/py | 240,916 |
| #3 | Prevalence String | epidemiology:ti OR prevalence:ti OR epidemiology:ab OR prevalence:ab AND [humans]/lim AND [english]/lim AND [abstracts]/lim AND [embase]/lim AND [2002-2012]/py | 180,556 |
| #4 | Cancer String | cancer*:ti OR carcinoma*:ti OR malignanc*:ti OR neoplasm*:ti OR cancer*:ab OR carcinoma*:ab OR malignanc*:ab OR neoplasm*:ab AND [humans]/lim AND [english]/lim AND [abstracts]/lim AND [embase]/lim AND [2002-2013]/py | 528,573 |
| #5 |  | #1 AND #2 AND #3 AND #4 | 568 |
| #6 | Not Reviews String | 'review'/exp OR review NOT (systematic OR 'meta analysis'/exp OR 'meta analysis' OR (indirect OR mixed AND 'treatment comparison')) AND [humans]/lim AND [english]/lim AND [abstracts]/lim AND [embase]/lim AND [2002-2013]/py | 583,239 |
| #7 |  | #5 NOT #7 | 443 |

We have also limited the search to not include non-systematic reviews published prior to 2002.

### Grey Literature Search

We searched the ‘grey’ literature (material that can be referenced but is not published in peer-reviewed, indexed medical journals) for any documents relating to the prevalence of HPV in head and neck cancers. We searched a variety of websites, identified relevant papers and screened them using the same inclusion criteria as for the identified medical journals:

- World Health Organization (WHO)
- Health Protection Agency (HPA)
- European Centre for Disease Control and Protection (ECDC)
- Northern Ireland Cancer Registry (NICR)
- Scottish Cancer Registry (Information Services Division, Scotland)
- Welsh Cancer Intelligence and Surveillance Unit (WCISU)
- Office of National Statistics (ONS).
