# Supplementary material for: Prevalence of human papillomavirus in head and neck cancers in European populations: a meta-analysis
Source: BMC Cancer. 2014 Dec 17;14:968. doi: 10.1186/1471-2407-14-968 (PMC4320477; doi:10.1186/1471-2407-14-968)
Supplement: Supplementary file 2 — Additional file 2: Table S1: Inclusion and Exclusion Criteria. Table S2. Modified Methodological Evaluation of Observation Research (MORE) Grading Criteria. Table S3. Characteristics of Included Studies. (DOCX 43 KB) [file 12885_2014_5178_MOESM2_ESM.docx]

# Additional file 2:

Table S1. Inclusion and Exclusion Criteria

| **Inclusion Criteria** | **Exclusion Criteria** |
| --- | --- |
| 1. *Patient population*  - Patients, or laboratory samples from patients, with head and neck cancer | 1. *Patient population*  - People (or laboratory samples) without head and neck cancer - Patients with only HPV infection, without head and neck cancer - Patients with human immunodeficiency virus (HIV) infection/acquired immunodeficiency syndrome (AIDS) co-morbidity |
| 1. *Outcomes*  - Specifically, the outcome to be extracted was: - Prevalence of the following HPV types (HPV-6, HPV-11, HPV-16, HPV-18, HPV-31, HPV-33, HPV-45, HPV-52, HPV-58) | 1. *Outcomes*  - HPV infection incidence rate only |
| 1. *Study design*    - Systematic reviews (including meta-analysis)    - Cohort studies    - Case-control studies    - Cross-sectional studies    - Randomised controlled trials  (if available) | 1. *Study design*  - Letters to the editor - Citations with no abstract - Case reports - Studies where the number of patients or samples analysed could not be determined |
| 1. *Year of study*  - Studies published between July 2002 and July 2012 |  |
| 1. *Languages*    - Only publications in English |  |
| 1. *Country*  - Only publications directly referring to, and quantifying prevalence data from, European populations |  |

Table S2. Modified Methodological Evaluation of Observation Research (MORE) Grading Criteria

| Instructions Please review the checklist and mark with an X quality terms that are reported, not reported and flaws in external or internal validity if present.  Descriptive Information  **Primary author name:**  **Year of study:**  **Reference:**  **Funding source:**  *Role of Funding Organisation in Data Analysis and Interpretations of the Results* | |
| --- | --- |
| Reported; sponsoring organisation was not involved in data analyses and interpretation of results | No flaw |
| Reported; sponsoring organisation (national funding body) was involved in data analyses and interpretation of results | Minor flaw |
| Reported; sponsoring organisation (manufacturer) was involved in data analyses and interpretation of results | Major flaw |
| Not reported | Major flaw |
| *Conflict of Interest* | |
| Reported; no conflict of interest | No flaw |
| Reported; conflict of interest present (if considered less likely to add bias) | Minor flaw |
| Reported; conflict of interest present (if authors paid directly by manufacturer) | Major flaw |
| Not reported | Major flaw |
| *Ethical Approval of the Study* | |
| Reported; study was approved by ethical committee | No flaw |
| Not reported | Major flaw |
| *Aim of the Study* | |
| Reported; included prevalence estimation with clear target population | No flaw |
| Reported; included prevalence estimation without clear target population | Minor flaw |
| Reported; did not include prevalence estimation with or without clear target population | Minor flaw |
| Not reported | Major flaw |
| *Study Design* | |
| Reported; prospective study | No flaw |
| Reported; retrospective study | Minor flaw |
| Reported; cross-sectional study | Minor flaw |
| Not reported; unclear | Major flaw |
| External Validity  **Sampling Bias**  *Sampling of the Subjects by the Investigators* | |
| Reported; random sampling of reference population | No flaw |
| Reported; convenient sampling of reference population | Minor flaw |
| Not reported; unclear | Major flaw |
| *Assessment of Sampling Bias by the Investigators* | |
| Reported; sampling bias was assessed by the authors – there were no differences in study population versus target population | No flaw |
| Reported; the authors did not assess sampling bias but justified exclusion of the subjects from the sampling or analysis | No flaw |
| Reported; sampling bias was assessed by the authors – there were differences in study population versus target population | No flaw |
| Reported; randomisation not described or mentioned | Minor flaw |
| Reported; the authors did not assess sampling bias | Minor flaw |
| Not reported; not conducted or unclear | Major flaw |
| **Estimate Bias**  *Exclusion Rate from the Analysis* | |
| Reported; ≤ 10% of recruited participants | No flaw |
| Reported; > 10% of recruited participants | Major flaw |
| Not reported; unclear | Major flaw |
| *Response Rate in Total Sample* | |
| Reported; > 60% of recruited participants | No flaw |
| Not applicable | No flaw |
| Reported; 40%–60% of recruited participants | Minor flaw |
| Reported; < 40% of recruited participants | Major flaw |
| Not reported; unclear | Major flaw |
| Internal Validity  *Source of Measure of Prevalence Described* | |
| Reported; measured objectively with diagnostic methods for the purpose of the study (independent of health care) | No flaw |
| Reported; obtained from registries or administrative databases (collected for epidemiological evaluation independent of health care) | Minor flaw |
| Reported; obtained from medical records (mining of data collected for health care purposes) | Minor flaw |
| Reported; obtained from hospital administrative databases (mining of data collected for health care purposes) | Minor flaw |
| Reported; proxy reported – collected from parents, relatives, etc. | Major flaw |
| Reported; self-reported (collected for the study) | Major flaw |
| Not reported; unclear | Major flaw |
| *Description of the Method of HPV Detection* | |
| Reported; detailed description of method of HPV-DNA detection (PCR, in situ hybridisation) | No flaw |
| Reported; detailed description of method of HPV-DNA detection not provided | Minor flaw |
| Not reported; unclear | Major flaw |
| *Reporting of Prevalence* | |
| Reported; period prevalence (prevalence of HPV over a period of time) | No flaw |
| Reported; point prevalence (prevalence of HPV on a certain date) | Minor flaw |
| Not reported; unclear | Major flaw |
| *Precision of Estimate (95% Confidence intervals, Standard deviations, Standard errors, etc.)* | |
| Reported | No flaw |
| Not reported; unclear | Minor flaw |

Table S3. Characteristics of Included Studies

| **Reference** | **Type of cancer** | **Country** | **Type of sample analysed** | **Type of primer used** | **Number of cancer samples evaluated** | **HPV6** | **HPV 11** | **HPV 16** | **HPV 18** | **HPV 31** | **HPV 33** | **HPV 45** | **HPV 52** | **HPV 58** |
| --- | --- | --- | --- | --- | --- | --- | --- | --- | --- | --- | --- | --- | --- | --- |
| Adamopoulou et al., 2008 | Oral | Germany and Greece | Exfoliated | MY09/11 | 68 | 7 | 0 | 1 | 3 | 1 | - | - | - | - |
| Anderson et al., 2007 | Laryngeal | Scotland | NR | GP5+6+ | 64 | 2 | - | - | - | - | - | - | - | - |
| Anderson et al., 2007 | Waldeyer's ring | Scotland | NR | GP5+6+ | 36 | 8 | - | 1 | 7 | - | - | - | - | - |
| Benevolo et al., 2011 | Oral | Italy | Fixed biopsy and exfoliated cells | NR | 20 | 1 | - | - | - | - | - | - | - | - |
| Benevolo et al., 2011 | Oropharyngeal | Italy | Fixed biopsy and exfoliated cells | NR | 11 | 5 | - | - | 5 | - | - | - | - | - |
| Christensen et al., 2011 | Tonsillar | Denmark | Fixed biopsy | NR | 58 | 37 | - | - | 35 | - | - | - | - | - |
| de Petrini et al., 2006 | Oral | Italy | Fixed biopsy | GP5+/GP6+ and MY09/MY11 | 23 | 9 | 0 | 0 | 9 | 0 | 0 | 0 | 0 | 0 |
| de Petrini et al., 2006 | Oropharyngeal | Italy | Fixed biopsy | GP5+/GP6+ and MY09/MY11 | 21 | 11 | 0 | 0 | 11 | 0 | 0 | 0 | 0 | 0 |
| DeVilliers et al., 2004 | Hypopharyngeal | Germany | Fixed biopsy | GP5+/GP6+, RS42/KM29, PC03/PC04, FAP | 1 | 1 | 1 | 1 | 0 | 0 | 0 | 0 | 0 | 0 |
| DeVilliers et al., 2004 | Laryngeal | Germany | Fixed biopsy | GP5+/GP6+, RS42/KM29, PC03/PC04, FAP | 3 | 3 | 0 | 0 | 2 | 0 | 0 | 0 | 0 | 0 |
| DeVilliers et al., 2004 | Oral | Germany | Fixed biopsy | GP5+/GP6+, RS42/KM29, PC03/PC04, FAP | 11 | 6 | 4 | 0 | 1 | 1 | 0 | 0 | 0 | 0 |
| DeVilliers et al., 2004 | Oropharyngeal | Germany | Fixed biopsy | GP5+/GP6+, RS42/KM29, PC03/PC04, FAP | 5 | 2 | 2 | 0 | 0 | 0 | 0 | 0 | 0 | 0 |
| Feher et al., 2009 | Oral | Hungary | Fixed biopsy | MY09/MY11, GP5+/GP6+ | 65 | 31 | - | - | - | - | - | - | - | - |
| Giovannelli et al., 2002 | Oral | Italy | Fixed biopsy | GP5+/GP6+ and MY09/MY11 | 13 | 8 | - | - | - | 7 | - | - | - | - |
| Giovannelli et al., 2006 | Oral | Italy | Exfoliated | GP5+/GP6+ and MY09/MY11 | 17 | 6 | - | - | - | - | - | - | - | - |
| Glombitza et al., 2010 | Laryngeal | Germany | Fixed biopsy | MY09/11 | 3 | 0 | - | - | 0 | - | - | - | - | - |
| Glombitza et al., 2010 | Paranasal sinus | Germany | Fixed biopsy | mY09/11 | 1 | 1 | - | - | 1 | - | - | - | - | - |
| Glombitza et al., 2010 | Pharyngeal | Germany | Fixed biopsy | mY09/11 | 5 | 1 | - | - | 1 | - | - | - | - | - |
| Glombitza et al., 2010 | Tonsillar | Germany | Fixed biopsy | mY09/11 | 29 | 22 | - | - | 22 | - | - | - | - | - |
| Gungor et al., 2007 | Laryngeal | Turkey | Fixed biopsy | Sp-10296/MP70215 | 95 | 7 | 2 | 7 | 1 | 0 | 0 | 0 | - | 0 |
| Hammarstedt et al., 2006 | Tonsillar | Sweden | Fixed biopsy | GP5+/GP6+, CPI/CPIIG | 203 | 99 | 0 | 0 | 86 | 0 | 0 | 3 | 1 | 0 |
| Hoffmann et al., 2005a | Hypopharyngeal | Germany | Fixed biopsy | MY09/11 | 8 | 4 | 0 | 0 | 4 | 0 | 0 | 0 | - | - |
| Hoffmann et al., 2005a | Laryngeal | Germany | Fixed biopsy | MY09/11 | 7 | 4 | 0 | 0 | 4 | 0 | 0 | 0 | - | - |
| Hoffmann et al., 2005a | Oral | Germany | Fixed biopsy | MY09/11 | 5 | 3 | 0 | 0 | 3 | 0 | 0 | 0 | - | - |
| Hoffmann et al., 2005a | Oropharyngeal | Germany | Fixed biopsy | MY09/11 | 3 | 1 | - | - | - | - | - | - | - | - |
| Hoffmann et al., 2005a | Tonsillar | Germany | Fixed biopsy | MY09/11 | 9 | 8 | 0 | 0 | 7 | 0 | 0 | 1 | - | - |
| Hoffmann et al., 2005b | Hypopharyngeal | Germany | Fixed biopsy | NR | 24 | 7 | - | - | 7 | - | - | - | - | - |
| Hoffmann et al., 2005b | Laryngeal | Germany | Fixed biopsy | NR | 19 | 5 | - | - | 5 | - | - | - | - | - |
| Hoffmann et al., 2005b | Oral | Germany | Fixed biopsy | NR | 6 | 4 | - | - | 4 | - | - | - | - | - |
| Hoffmann et al., 2005b | Oropharyngeal | Germany | Fixed biopsy | NR | 4 | 1 | - | - | 1 | - | - | - | - | - |
| Hoffmann et al., 2005b | Tonsillar | Germany | Fixed biopsy | NR | 20 | 11 | - | - | 10 | - | - | 1 | - | - |
| Jalouli et al., 2012 | Oral | Norway, Sweden, UK | Fixed biopsy | MY09/MY11 | 57 | 21 | - | - | - | - | - | - | - | - |
| Kansky et al., 2003 | Oral | Slovenia | Fixed biopsy | PGMY09/PGMY11, GP5+/GP6+ and WD72/WD76/WD66/WD154, and KM29/RS42 | 35 | 1 | 0 | 0 | 0 | 0 | 0 | 1 | 0 | 0 |
| Kansky et al., 2003 | Oropharyngeal | Slovenia | Fixed biopsy | PGMY09/PGMY11, GP5+/GP6+ and WD72/WD76/WD66/WD154, and KM29/RS42 | 4 | 2 | 0 | 0 | 2 | 0 | 0 | 0 | 0 | 0 |
| Kansky et al., 2003 | Tongue | Slovenia | Fixed biopsy | PGMY09/PGMY11, GP5+/GP6+ and WD72/WD76/WD66/WD154, and KM29/RS42 | 23 | 2 | 0 | 0 | 1 | 0 | 0 | 0 | 0 | 0 |
| Kaschke et al., 2011 | Base of tongue | Unclear | NR | NR | 54 | 29 | - | - | - | - | - | - | - | - |
| Kaschke et al., 2011 | Oropharyngeal | Unclear | NR | NR | 114 | 73 | - | - | - | - | - | - | - | - |
| Kaschke et al., 2011 | Tonsillar | Unclear | NR | NR | 60 | 44 | - | - | - | - | - | - | - | - |
| Klozar et al., 2008 | Base of tongue | Czech Republic | Fixed biopsy | GP5+/GP6+ | 10 | 5 | - | - | - | 0 | 0 | - | 0 | 0 |
| Klozar et al., 2008 | Tonsillar | Czech Republic | Fixed biopsy | GP5+/GP6+ | 51 | 41 | - | - | - | 0 | 0 | - | 0 | 0 |
| Klussman et al., 2003 | Tonsillar | Germany | Fixed biopsy | A10/A5-A6/A8, CP62/70-CP65/69a | 34 | 18 | - | - | 17 | - | - | 1 | - | - |
| Knoedler et al., 2011 | Base of tongue | Germany | NR | NR | 54 | 29 | - | - | - | - | - | - | - | - |
| Knoedler et al., 2011 | Tonsillar | Germany | NR | NR | 60 | 44 | - | - | - | - | - | - | - | - |
| Koskinen et al., 2003 | Hypopharyngeal | Finland | Fixed biopsy | SPF10, FAP 59/64, CP65/70, CP 66/69, INNO-LiPA | 10 | 5 | 1 | 0 | 4 | 0 | 0 | 2 | 0 | 0 |
| Koskinen et al., 2003 | Laryngeal | Finland | Fixed biopsy | SPF10, FAP 59/64, CP65/70, CP 66/69, INNO-LiPA | 18 | 9 | 2 | 1 | 9 | 0 | 0 | 2 | 0 | 1 |
| Koskinen et al., 2003 | Oral | Finland | Fixed biopsy | SPF10, FAP 59/64, CP65/70, CP 66/69, INNO-LiPA | 13 | 7 | 0 | 0 | 6 | 0 | 0 | 0 | 0 | 0 |
| Koskinen et al., 2003 | Tongue | Finland | Fixed biopsy | SPF10, FAP 59/64, CP65/70, CP 66/69, INNO-LiPA | 15 | 11 | 0 | 0 | 7 | 0 | 0 | 6 | 0 | 0 |
| Koskinen et al., 2003 | Tonsillar | Finland | Fixed biopsy | SPF10, FAP 59/64, CP65/70, CP 66/69, INNO-LiPA | 5 | 5 | 0 | 0 | 5 | 0 | 0 | 0 | 0 | 0 |
| Koskinen et al., 2007 | Laryngeal | Finland, Norway, Sweden | Fixed biopsy | MY09/11 and GP5+/6+ and SPF10 | 69 | 3 | 0 | 0 | 1 | 0 | 0 | 0 | 0 | 0 |
| Licitra et al., 2006 | Oropharyngeal | Italy | Fixed biopsy | NR | 90 | 17 | - | - | 17 | 0 | - | - | - | - |
| Lindel et al., 2009 | Oropharyngeal | Germany | Fixed biopsy | GP5+/GP6+, FAP, SPF, CP | 20 | 4 | - | - | - | - | - | - | - | - |
| Lindel et al., 2009 | Tonsillar | Germany | Fixed biopsy | GP5+/GP6+, FAP, SPF, CP | 9 | 6 | - | - | - | - | - | - | - | - |
| Lopes et al., 2011 | Oral | United Kingdom | Fixed biopsy | GP5+6+ | 142 | 2 | 0 | 0 | 2 | 0 | 0 | 0 | 0 | 0 |
| Major et al., 2005 | Pharyngeal | Hungary | Fixed biopsy | MY09/11 and GP5+/6+ | 5 | 1 | 1 | 0 | 0 | 0 | 0 | 0 | 0 | 0 |
| Mellin et al., 2002 | Tonsillar | Sweden | Fixed biopsy | GP5+/GP6+, CPI/CPIIG | 22 | 12 | 0 | 0 | 11 | 0 | 0 | 1 | 0 | 0 |
| Morshed et al., 2008 | Laryngeal | Poland | Fixed biopsy | INNO LiPA SPF10 | 93 | 33 | 0 | 0 | 28 | 6 | 0 | 5 | 0 | 0 |
| Nemes et al., 2006 | Oral | Hungary | Fixed biopsy | MY09/MY11 | 79 | 33 | - | - | 27 | - | - | - | - | - |
| Reimers et al., 2007 | Oropharyngeal | Germany | Fixed biopsy | A10/A5-A6/A8; CP62/70-CP65/69 | 106 | 30 | - | - | 29 | - | - | 1 | - | - |
| Scapoli et al., 2009 | Oral | Italy | Fixed biopsy | NR | 314 | 5 | - | - | 5 | 0 | 0 | - | - | - |
| St Guily et al., 2011 | Base of tongue | France | Fixed biopsy | INNO-LiPA | 43 | 13 | - | - | - | - | - | - | - | - |
| St Guily et al., 2011 | Oral | France | Fixed biopsy | INNO-LiPA | 209 | 22 | - | - | 21 | - | - | - | - | - |
| St Guily et al., 2011 | Oropharyngeal | France | Fixed biopsy | INNO-LiPA | 314 | 146 | - | - | 131 | - | - | - | - | - |
| Szarka et al., 2009 | Oral | Hungary | Fixed biopsy | MY09/11 and GP5+/6+ | 65 | 31 | 0 | 4 | 18 | 4 | 1 | 2 | 0 | 0 |
| Tachezy et al., 2005 | Oropharyngeal | Czech Republic | Fixed biopsy | GP5+/GP6+ and PC03/04 | 68 | 35 | 0 | 0 | 28 | 0 | 0 | 3 | 0 | 0 |
| Tachezy et al., 2009 | Oropharyngeal | Czech Republic | Fixed biopsy | GP5+/GP6+ and MY09/11 | 86 | 53 | - | - | 49 | - | - | 3 | - | - |
| Van Doornum et al., 2003 | Laryngeal | The Netherlands | Serum | NR | 127 | 25 | - | - | 25 | - | - | - | - | - |
| Van Doornum et al., 2003 | Oropharyngeal | The Netherlands | Serum | NR | 48 | 16 | - | - | 16 | - | - | - | - | - |
| Van Doornum et al., 2003 | Tongue | The Netherlands | Serum | NR | 56 | 12 | - | - | 12 | - | - | - | - | - |
| Weiss et al., 2011 | Base of tongue | Germany | Fixed biopsy | HPV16 E6, Primer 1, forward; HPV16 E6, Primer 1, reverse; HPV16 E7, Primer 1, forward; HPV16 E7, Primer 1, reverse | 32 | 16 | - | - | 16 | - | - | - | - | - |
| Weiss et al., 2011 | Hypopharyngeal | Germany | Fixed biopsy | HPV16 E6, Primer 1, forward; HPV16 E6, Primer 1, reverse; HPV16 E7, Primer 1, forward; HPV16 E7, Primer 1, reverse | 7 | 0 | - | - | 0 | - | - | - | - | - |
| Weiss et al., 2011 | Oral | Germany | Fixed biopsy | HPV16 E6, Primer 1, forward; HPV16 E6, Primer 1, reverse; HPV16 E7, Primer 1, forward; HPV16 E7, Primer 1, reverse | 15 | 0 | - | - | 0 | - | - | - | - | - |
| Weiss et al., 2011 | Pharyngeal | Germany | Fixed biopsy | HPV16 E6, Primer 1, forward; HPV16 E6, Primer 1, reverse; HPV16 E7, Primer 1, forward; HPV16 E7, Primer 1, reverse | 3 | 1 | - | - | 1 | - | - | - | - | - |
| Weiss et al., 2011 | Pharyngeal | Germany | Fixed biopsy | HPV16 E6, Primer 1, forward; HPV16 E6, Primer 1, reverse; HPV16 E7, Primer 1, forward; HPV16 E7, Primer 1, reverse | 12 | 1 | - | - | 1 | - | - | - | - | - |
| Weiss et al., 2011 | Tongue | Germany | Fixed biopsy | HPV16 E6, Primer 1, forward; HPV16 E6, Primer 1, reverse; HPV16 E7, Primer 1, forward; HPV16 E7, Primer 1, reverse | 19 | 1 | - | - | 1 | - | - | - | - | - |
| Weiss et al., 2011 | Tonsillar | Germany | Fixed biopsy | HPV16 E6, Primer 1, forward; HPV16 E6, Primer 1, reverse; HPV16 E7, Primer 1, forward; HPV16 E7, Primer 1, reverse | 45 | 17 | - | - | 17 | - | - | - | - | - |
| Weiss et al., 2011 | Waldeyer's ring | Germany | Fixed biopsy | HPV16 E6, Primer 1, forward; HPV16 E6, Primer 1, reverse; HPV16 E7, Primer 1, forward; HPV16 E7, Primer 1, reverse | 77 | 33 | - | - | 33 | - | - | - | - | - |

HPV: human papillomavirus; NR: not reported
